# Effect of a Tongue Training Device on Tongue Strength in Obstructive Sleep Apnea Patients with Varying Degrees of Tongue Base Collapse by DISE Undergoing Modified Uvulopalatopharyngoplasty

**DOI:** 10.3390/healthcare13192509

**Published:** 2025-10-02

**Authors:** Yung-An Tsou, Hsueh-Hsin Kao, Ya-Han Lin, Yu-Jen Chou, Yee-Hsin Kao, Jui-Kun Chiang

**Affiliations:** 1 Department of Otorhinolaryngology-Head and Neck Surgery, China Medical University Hospital, Taichung 404, Taiwan; d22052121@gmail.com (Y.-A.T.); rita208911@gmail.com (Y.-H.L.); 2 Department of Otolaryngology Head and Neck Surgery, Asia University Hospital, Taichung 413, Taiwan; 3 School of Medicine, China Medical University, Taichung 404, Taiwan; 4 Department of Audiology and Speech-Language Pathology, Asia University, Taichung 413, Taiwan; 5 Department of Radiation Oncology, Taichung Veterans General Hospital, Taichung 407, Taiwan; kaogrady8176@gmail.com; 6 Nature Dental Clinic, Nantou 545, Taiwan; webbchou1220@gmail.com; 7 Department of Family Medicine, Tainan Municipal Hospital (Managed by Show Chwan Medical Care Corporation), Tainan 701, Taiwan; 8 Department of Family Medicine, Dalin Tzu Chi Hospital, Buddhist Tzu Chi Medical Foundation, Chiayi 622, Taiwan

**Keywords:** Iowa Oral Performance Instrument (IOPI), tongue, strength, obstructive sleep apnea (OSA)

## Abstract

(1) **Background:** The Iowa Oral Performance Instrument (IOPI) is the well-established device for assessing tongue strength. The current study aimed to assess the effectiveness of the HEAL device in patients with obstructive sleep apnea (OSA) exhibiting varying degrees of tongue base obstruction, as determined by drug-induced sleep endoscopy (DISE). All participants had undergone modified uvulopalatopharyngoplasty (UPPP), and tongue strength was measured using the IOPI. (2) **Methods:** This retrospective observational study utilized DISE to assess patterns of upper airway collapse in patients with OSA who were candidates for surgical intervention. Based on DISE findings, patients were divided into two groups: the M group (no or partial tongue base obstruction) and the S group (severe obstruction). The first tongue strength assessment using the IOPI was conducted one month after modified UPPP, prior to initiating HEAL training. Patients then underwent tongue muscle training with the HEAL device, starting one-month post-surgery. A second IOPI assessment was performed after at least one month of training. (3) **Results:** Forty-nine OSA patients with varying degrees of tongue base obstruction (assessed via DISE) received modified UPPP and were included in the analysis. The mean age was 38.3 ± 7.4 years, and mean BMI was 27.8 ± 3.9 kg/m^2^. After training with the HEAL, average tongue strength increased by 20.6 ± 11.5 kPa. The M group showed significantly greater improvement than the S group (22.45 ± 11.77 vs. 13.33 ± 6.93 kPa, *p* = 0.024). Linear regression confirmed this difference remained significant (*p* = 0.024). (4) **Conclusions:** In this study, participants who received modified UPPP exhibited improvements in tongue base strength following a minimum of one month of training with the HEAL device. Those with no or mild tongue base obstruction, as determined by DISE prior to surgery, experienced greater improvements in tongue strength compared to those with severe tongue base obstruction.

## 1. Introduction

Obstructive sleep apnea (OSA) is a significant sleep disorder associated with reduced quality of life, as well as an increased risk of hypertension and both cardiovascular and cerebrovascular diseases [1]. According to a systematic review, the mean prevalence of OSA, as defined by an apnea–hypopnea index (AHI) of ≥5, was 22% in men and 17% in women between 1993 and 2013 [2]. OSA is associated with a wide range of adverse health outcomes. If left untreated, it can result in excessive daytime sleepiness, cognitive impairment, reduced work performance, and a diminished health-related quality of life. As such, OSA represents a major public health concern and is strongly linked to the development of several serious conditions, including hypertension [3,4], type 2 diabetes [5], stroke [6,7], congestive heart failure [8], coronary artery disease [9,10,11], and cardiac arrhythmias [12]. Moreover, untreated OSA is associated with an elevated risk of early mortality [13,14].

Continuous Positive Airway Pressure (CPAP) is the recommended first-line management for individuals with OSA [15]. Nevertheless, CPAP adherence rates vary widely—an estimated 17% to 85% of patients fail to comply, leaving a substantial portion of individuals effectively untreated [16]. This wide gap in treatment adherence highlights the critical need for alternative or adjunctive therapeutic options that are more acceptable and sustainable for patients. As a result, these patients often seek surgical treatment for their condition. Various surgical procedures are employed to treat OSA in adults, which include maxillomandibular advancement (MMA), pharyngeal surgeries such as uvulopharyngopalatoplasty (UPPP), as well as multi-level and staged surgical approaches. A recent systematic review and meta-analysis reported a 33% reduction in mean AHI (from 40.3 to 29.8 events per hour) observed with UPPP alone [17].

Modified UPPP has been demonstrated to be an effective long-term treatment option for individuals with OSA who are appropriate candidates for surgical intervention [18]. Previous reviews have emphasized the value of drug-induced sleep endoscopy (DISE) in evaluating the anatomical characteristics of OSA [19]. DISE is commonly employed to evaluate upper airway dynamics, identify both primary and secondary sites of obstruction, and guide the selection of appropriate surgical approaches in adults and children with OSA. [20]. For patients considering surgical treatment, such as modified UPPP, DISE serves as a valuable preoperative tool to evaluate the location and pattern of sleep-related upper airway collapse. It is specifically designed to assess the nature of upper airway obstruction during a sleep-like state and to inform a tailored treatment strategy. Following the DISE examination, the surgeon evaluates the sites of upper airway collapse using the VOTE classification system—velum (V), oropharyngeal lateral walls (O), tongue base (T), and epiglottis (E)—which is the most widely adopted scoring system for DISE assessments [21]. According to the criteria described by Kezirian, the severity of obstruction is classified as follows: 0—no obstruction (no vibration or <50% narrowing), 1—partial obstruction (vibration or 50–75% narrowing), and 2—complete obstruction (collapse or >75% narrowing) [21]. Posterior displacement and collapse at the tongue base are common in OSA; using the VOTE classification, multilevel collapse occurs in ≈68–76% of cases, underscoring the clinical relevance of tongue position and strength [22].

Traditional oral strength therapies encompass a range of established techniques, including oromotor sensory stimulation [23], the Mendelsohn maneuver [24], supraglottic swallowing [23], range-of-motion exercises, oromotor strengthening [25], chin-down/chin-tuck positioning [26], head turning toward the weaker side, tilting toward the stronger side [23], effortful swallowing [27], and cough training [28]. Additional interventions include tongue strengthening exercises [29] and isometric lingual exercise programs [30]. Among the tools developed to measure and enhance tongue strength is the IOPI [31]. In this study, we introduced the HEAL—a tongue-strengthening device made of medical-grade silicone—specifically designed to improve tongue muscle function through a combination of passive tongue depression and active tongue-pressing exercises. Tongue strength may decrease after modified UPPP due to postoperative pain or other complications. However, it may return to baseline approximately one month after surgery [32].

The initial tongue strength assessment (baseline measurement), using the IOPI, was conducted one month after modified UPPP and prior to the initiation of HEAL training. Patients then began tongue muscle training with the HEAL device starting at least one-month post-surgery. A second IOPI assessment was conducted following a minimum of one month of training. We hypothesized that tongue strength would improve following tongue muscle training with the HEAL device. The aim of this current study was to evaluate the effectiveness of the HEAL in patients with OSA who demonstrated varying degrees of tongue base obstruction, as assessed by DISE. Tongue strength was quantitatively measured utilizing the IOPI.

## 2. Materials and Methods

### 2.1. Study Design

This retrospective observational study aimed to evaluate the effect of the HEAL device on tongue strength in patients with OSA who exhibited varying degrees of tongue base obstruction following modified UPPP. Tongue strength was measured using the IOPI. The study protocol was reviewed and approved by the Institutional Review Board of the Research Ethics Committee at China Medical University Hospital, Taiwan (Approval No. CMUH 110-REC3-200).

### 2.2. Study Population

Patients with OSA sought treatment at the ENT department of a tertiary hospital in Taiwan between 7 March 2022, and 27 February 2023. Written informed consent was obtained from all participants prior to enrollment. The inclusion criterion for this study was OSA patients who began using the HEAL device at least one month after undergoing modified UPPP. The exclusion criterion was patients who, despite meeting the inclusion criteria, were unable to tolerate tongue muscle training with the HEAL device.

### 2.3. Interventions

**Figure 1:**
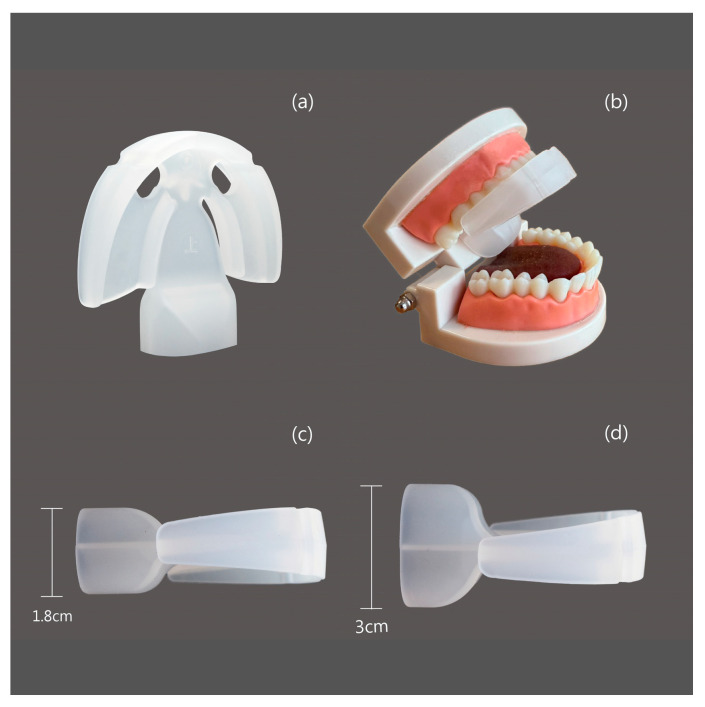
The configuration of the HEAL, a tongue-strengthening tool. (**a**) The vertical configuration; (**b**) the configuration situated in the dental mold; (**c**) the Basic configuration, measuring 5.5 × 6.0 × 1.8 cm; and (**d**) the Enhanced configuration, measuring 5.5 × 6.0 × 3.0 cm.

**Figure 2:**
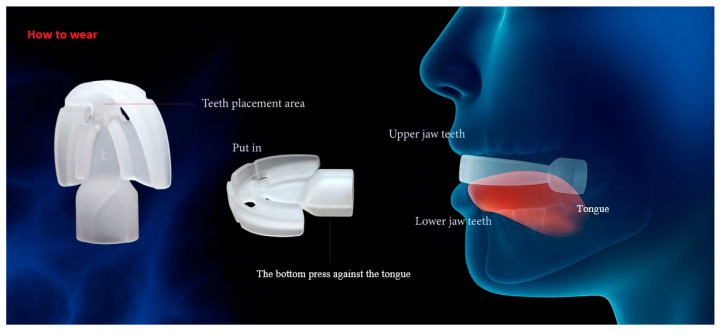
Instructions for using the HEAL are demonstrated.

We introduced a tongue-strengthening device called the HEAL, made of medical-grade silicone and designed to enhance tongue strength. Each patient received a HEAL device (Patent No. M587995, Taiwan) [33] (Figure 1). The HEAL device is made of medical-grade silicone, with material specifications compliant with ISO 10993-5, ISO 10993-23, ISO 10993-10, and ISO 10993-12 standards [34,35,36,37], and is free of latex and BPA. It is designed to enhance tongue strength through both passive tongue depression and active tongue-pressing exercises. Participants were all instructed to begin utilizing the HEAL one month after receiving modified UPPP for OSA, as this delay helps reduce the risk of postoperative wound bleeding. The HEAL is intended for use during waking hours and is not recommended during sleep. When worn, the tongue is positioned beneath the device and combination of passive tongue depression and active tongue-pressing exercises. Each training session lasts approximately 5–10 min, with a total recommended daily usage time of 30–40 min. The procedure for using the HEAL device is illustrated in Figure 2. In this study, tongue strength was assessed using the Iowa Oral Performance Instrument (IOPI, Model 2.2; IOPI Medical LLC, Carnation, WA, USA), which consists of a plastic catheter connected to an air-filled balloon. During the assessment, participants were instructed to sit in a relaxed position and apply maximum pressure to the balloon by pressing their tongue against it for three seconds. Tongue strength was specifically evaluated during tongue elevation, with emphasis on the posterior region of the tongue. The IOPI device was calibrated prior to each measurement. Both baseline and follow-up assessments consisted of three trials, and the average of the three measurements was used for analysis. The first tongue strength assessment using the IOPI was conducted one month after modified UPPP, prior to initiating HEAL training. Patients then underwent tongue muscle training with the HEAL device, starting one-month post-surgery. A second IOPI assessment was performed after at least one month of training.

### 2.4. Outcome Measures

The objective of this study was to evaluate and compare tongue strength before and after at least one month use of the HEAL device in patients with OSA who had undergone modified UPPP, stratified by the severity of tongue base obstruction, as determined by DISE. Patients with no or partial tongue base obstruction were categorized into the M group, while those with severe obstruction were assigned to the S group. Tongue strength was measured using the IOPI.

### 2.5. Statistical Analysis

All statistical analyses were conducted using R software (version 4.4.1; R Foundation for Statistical Computing, Vienna, Austria). A two-tailed *p*-value < 0.05 was considered indicative of statistical significance. Categorical variables are presented as frequencies and percentages, and continuous variables are reported as means ± standard deviations; in bar plots, data are shown as means ± standard errors. Shapiro–Wilk test was used for normality test. Depending on the data distribution, comparisons of continuous variables between two groups were conducted using the *t*-test, Fisher’s exact test, or Wilcoxon rank-sum test. Smoothing curves were plotted to illustrate the trends. Linear regression analysis was conducted to identify significant factors associated with the outcome. Bar plots were used to illustrate comparisons between the two groups. With a sample size of 49 and α = 0.10, the estimated power to detect the prespecified effect size in a single-predictor model was 74.37%.

## 3. Results

**Figure 3:**
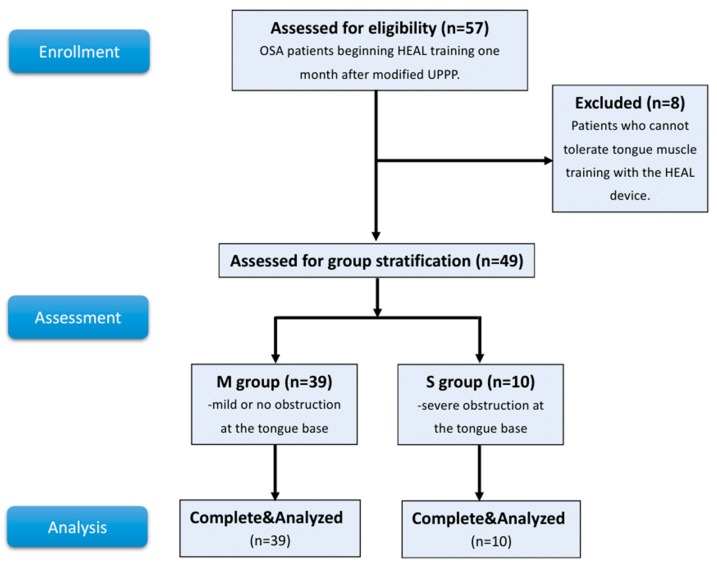
Flow chart of the current study.

A total of 57 patients with OSA, presenting varying degrees of tongue base obstruction as assessed by DISE, underwent modified UPPP and were initially included. Eight patients were excluded due to intolerance to tongue muscle training—six for daily use <30 min, one for oral discomfort, and one for persistent nausea—resulting in 49 patients included in the final analysis. The study flowchart is presented in Figure 3.

The improvement in tongue strength following HEAL device (Figure 1) use was significantly greater in patients with mild or no tongue base obstruction (M group) compared to those with severe obstruction (S group). The mean age of the study population was 38.3 ± 7.44 years, and the mean BMI was 27.8 ± 3.9 kg/m^2^ (Table 1). There were no significant differences between the M and S groups in terms of AHI, preoperative IOPI score, and number of training days (Table 2).

In this study, the average improvement in tongue strength following HEAL training was 20.6 ± 11.5 kPa, corresponding to an approximate 74.6% increase from the baseline measurement. The M group demonstrated a significantly greater improvement compared to the S group (22.45 ± 11.77 kPa vs. 13.33 ± 6.93 kPa, *p* = 0.024) (Table 2). The statistical power of this analysis was 0.884 with an alpha level of 0.05. Patients with less tongue base collapse following modified UPPP surgery tend to show greater improvement in tongue muscle strength after training with the HEAL device.

**Figure 4:**
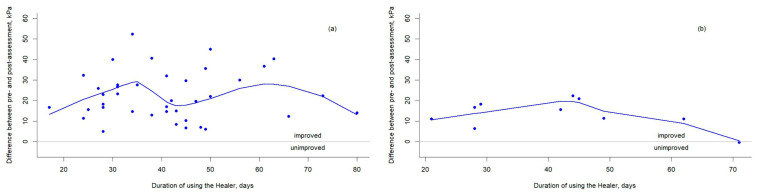
The scatter plots and smoothing lines show the differences in tongue base obstruction severity levels between pre- and post-assessments plotted against training days for (**a**) the M group and (**b**) the S group.

Scatter plots with smoothing lines are shown in Figure 4. In Figure 4a, patients in the M group demonstrated an improvement in tongue muscle strength of 22.5 ± 11.8 kPa after 41.3 ± 14.1 days of training, with the greatest improvement observed within the first 35 days of HEAL-based tongue muscle training. In Figure 4b, patients in the S group showed an improvement of 13.3 ± 6.9 kPa after 41.9 ± 16.0 days of training, with the peak improvement occurring within the first 45 days of training with the HEAL device. Univariate linear regression analysis revealed that among the variables examined—gender, age, BMI, smoking history, alcohol consumption, history of hypertension, history of diabetes, training days, and group classification—only group classification reached statistical significance. Patients in the M group demonstrated an average improvement in tongue strength that was 9.12 kPa greater than that of the S group (*p* = 0.024) (Table 3).

**Figure 5:**
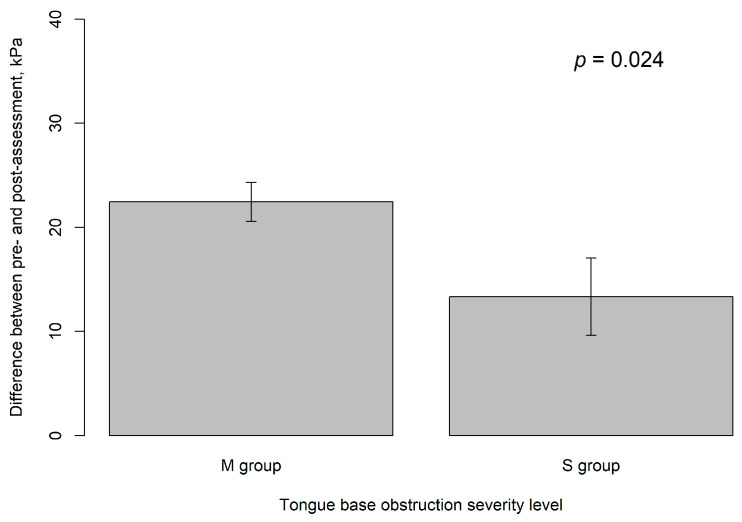
The bar plots compare the severity levels of tongue base obstruction between the M group and the S group, as measured by IOPI while using the HEAL device.

In the multivariate linear regression analysis, we evaluated Models 1 through 6 to identify significant factors associated with the improvement in tongue muscle strength from pre-operation to post-training with the HEAL device. Model 1 included the covariates: group (M vs. S), gender, age, BMI, smoking history, and training days. No significant predictors were identified in this model. We then progressively reduced the number of covariates from Models 2 to 5, but none of these models yielded statistically significant results. In Model 6, participants in the M group showed a significantly greater improvement in tongue strength after undergoing modified UPPP and HEAL training compared to those in the S group (*p* = 0.024). Gender and the number of training days were not found to be significant predictors (Table 4). Figure 5 presents bar plots comparing the severity of tongue base obstruction between the M and S groups, showing that patients in the M group had significantly greater improvement in tongue strength than those in the S group (*p* = 0.024), based on IOPI measurements with the HEAL device.

## 4. Discussion

Our study demonstrates that tongue muscle training with the HEAL device, initiated one month after modified UPPP, resulted in a significant increase in tongue strength. The mean improvement of 20.6 ± 11.5 kPa, representing a 74.6% gain, indicates that most participants not only recovered their baseline strength but also achieved substantial enhancement beyond it. Participants with no or mild tongue base obstruction, as assessed by DISE prior to surgery, exhibited greater improvements in tongue base strength compared to those with severe obstruction. Additionally, our findings suggest that lower severity of tongue anteroposterior (AP) obstruction—based on the VOTE scoring system evaluated during DISE—is associated with greater gains in tongue strength following HEAL device training. Clinically, patients with less tongue base collapse following modified UPPP surgery tend to exhibit greater improvements in tongue muscle strength after training with the HEAL device.

The human tongue is composed of two primary types of muscle fibers. The posterior portion is primarily composed of type I slow-twitch fibers, which facilitate sustained tonic activities, including the maintenance of retroglossal airway patency [38,39]. In contrast, the anterior tongue is composed predominantly of type II fast-twitch fibers, which enable brief, high-intensity activities such as chewing and speaking [40]. Therefore, the posterior part of the tongue plays a critical role in swallowing and maintaining airway patency.

A previous review reported that DISE is an effective tool to evaluate the anatomical features of OSA [19]. Another study demonstrated that anterior–posterior collapse of the tongue base is significantly correlated with the severity of OSA [41]. DISE provides valuable information for patient management [42] and enhances treatment planning for patients with OSA [43], particularly when surgical intervention is being considered. Another previous study reported that the most commonly identified site of obstruction in DISE scoring systems was the tongue base (62%), followed by the lateral pharynx/oropharynx (57%), palate (57%), epiglottis/supraglottis (38%), and hypopharynx (38%) [20]. These sites were evaluated using the VOTE classification, which includes assessment of velum, oropharyngeal lateral walls, tongue base, and epiglottis.

Compared with previous studies—where tongue-strengthening exercise interventions resulted in an increase of 8.12 kPa in posterior tongue strength [29], isometric lingual exercise programs led to an 80.8% improvement [30], and the use of the IOPI showed a 14.0% improvement—the current study demonstrated a greater absolute increase of 20.6 ± 11.5 kPa, corresponding to an approximate 74.6% improvement after HEAL use. These differences may be attributed to variations in study populations and protocols. Robbins et al. and Park et al. studied stroke patients with dysphagia [30,31], while Lin et al.’s study [29] focused on healthy individuals. In contrast, this study examined OSA patients who had undergone modified UPPP. The training durations also differed: 8 weeks in Lin et al. and Robbins et al., 4 weeks in Park et al., and a minimum of one month in the present study. In this study, we observed a mean increase of 20.6 ± 11.5 kPa in posterior tongue pressure after HEAL use among patients with obstructive sleep apnea (OSA) who had undergone modified UPPP. On this basis, HEAL may be considered as an adjunctive post-UPPP therapy in selected patients with OSA. Given the posterior tongue’s role in providing sustained tonic activity to maintain retroglossal airway patency, this pressure gain may indirectly indicate improved airway stability. However, the apnea–hypopnea index (AHI) was not measured after HEAL use; therefore, a direct effect on OSA severity cannot be inferred. Prospective studies incorporating post-intervention AHI and other clinical outcomes are warranted to confirm efficacy.

Recent studies reinforce the value of combining structural airway modification with tongue strengthening in OSA management. A 2024 meta-analysis of myofunctional therapy confirmed significant improvements in tongue elevation motor skills [44]. A 2023 investigation found that MMA plus genioglossus advancement (GA) led to anterior repositioning of the hyoid and tongue—with improvements correlating with sustained AHI reduction one-year post-op [45]. Similarly, a 2022 study using modified GA and radiofrequency tongue-base reduction documented marked enhancement in airway patency and tongue-base posture at six months [46]. Building upon prior research and our present findings, this underscores those structural modifications—whether surgical or device-based—paired with targeted tongue exercises provide synergistic benefits in stabilizing the upper airway in patients with OSA.

In the current study, particular emphasis was placed on assessing tongue base obstruction using the VOTE classification. This focus may have stemmed from the physician’s belief that tongue base obstruction is closely associated with the severity of OSA. However, this represents a limitation, as the DISE findings were not comprehensively evaluated across all VOTE components. Additionally, while the IOPI is a reliable device to measure tongue strength for patients with OSA [47], we assessed only the posterior tongue. This choice was based on pathophysiological relevance, since posterior tongue function is closely linked to tongue-base collapse and upper-airway patency and is routinely contextualized within the VOTE/DISE framework [21]. Moreover, posterior placement yields more reproducible measurements [48]. Nevertheless, anterior and posterior regions differ systematically (with anterior pressures typically higher) [49], so our findings cannot be extrapolated to anterior-tongue performance. Future studies should incorporate both anterior and posterior assessments for a more comprehensive evaluation.

Overall, our study demonstrated that tongue strength significantly improved in OSA patients following a minimum of one month training period with the HEAL device after undergoing modified UPPP. Notably, patients with severe tongue base obstruction showed less improvement compared to those with no or mild obstruction, suggesting that anatomical factors may influence the effectiveness of tongue muscle training. This finding underscores the importance of targeted tongue base strengthening in the management of OSA, particularly after surgical interventions such as modified UPPP. The clinical relevance of the observed improvement lies in the potential enhancement of upper airway muscle tone, which may help reduce airway collapse during sleep. Beyond pathophysiology, randomized evidence shows that myofunctional therapy improves OSA metrics (e.g., AHI), supporting the rationale for assessing and training tongue function [50]. Alongside, existing literature indicates that therapies targeting tongue positioning—including orofacial myofunctional therapy, oral appliances, daytime neuromuscular stimulation, and hypoglossal nerve stimulation—can improve OSA severity and patient-reported outcomes [51,52,53]. However, given the suboptimal adherence rates to CPAP therapy, the HEAL device may serve as a supportive, non-invasive adjunct, especially for post-surgical patients or those with minimal tongue base involvement. Nevertheless, a prospective case–control study is warranted to investigate the effect of the HEAL device on tongue muscle strength in patients undergoing modified UPPP surgery.

This study has several limitations. First, as a retrospective study, tongue base strength was not assessed prior to the modified UPPP procedure. Future prospective studies are needed to address this issue. The retrospective design also limits control over the timing between surgery, initiation of HEAL use, and IOPI assessments; standardized peri-operative timelines and pre-specified stratification by collapse pattern (e.g., DISE) should be incorporated prospectively. Second, the sample size in the final analysis was relatively small, comprising only 49 patients (39 males and 10 females). The power analysis in the current study yielded a value of 74.37%. This level of power may be considered acceptable for exploratory or pilot studies conducted under resource constraints. Accordingly, the findings should be interpreted with caution and warrant validation in larger, adequately powered cohorts in future confirmatory research. Third, data from 8 patients were unavailable due to intolerance to the HEAL device; these cases were excluded from the analysis, resulting in a final cohort of 49 patients. This complete-case approach may introduce attrition bias if missingness was not completely at random. Prospective protocols for related studies in the future should predefine retention strategies, objective scheduling windows, and missing-data handling (e.g., multiple imputation or sensitivity analyses), and report CONSORT-style flow to enhance transparency. Additionally, further research is necessary to develop a standardized training protocol for the use of the HEAL device. Such a protocol should specify frequency, session duration, progression criteria, warm-up routines, minimum adherence thresholds, and peri-operative timing to reduce heterogeneity and improve reproducibility. Fourth, compliance was monitored through weekly telephone interviews rather than in-person supervision. Self-report is vulnerable to recall and social-desirability biases, and we did not employ device-based telemetry or electronic logs to validate training time. In addition, some participants experienced transient nausea during the initial stages of using the HEAL device, which may have affected adherence. Performing tongue warm-up exercises prior to device use may help alleviate this early discomfort. Future studies should capture tolerability using a standardized scale, record all adverse events systematically, and evaluate whether structured warm-up meaningfully improves early adherence. Fifth, the detraining effect—defined as the decline in tongue muscle strength following cessation of training—was not evaluated in this study. Durability and maintenance dosing therefore remain unknown; longitudinal follow-up with planned off-training intervals (e.g., 1–3 months) and predefined maintenance schedules would clarify the trajectory of strength retention. Finally, while our findings suggest that the HEAL device may be effectively combined with surgical treatments for OSA to enhance posterior tongue strength, its direct impact on OSA-related outcomes (such as AHI, daytime symptoms, or quality of life) remains to be clarified and warrants further investigation to better establish the clinical relevance of tongue training devices in OSA management. Future trials should include PSG-based endpoints (AHI/ODI), validated symptom and quality-of-life measures, assessor blinding where feasible, and comparator arms (e.g., sham or alternative active adjuncts) to strengthen causal inference. Additionally, the single-center design may limit external validity; multi-center studies across diverse care settings are needed.

## 5. Conclusions

In the current study, participants who underwent modified UPPP improved tongue base strength after receiving the HEAL with a minimum one-month period of training. On average, tongue base strength increased by 20.6 ± 11.5 kPa, corresponding to an approximate 74.6% improvement from baseline. Participants with no or mild tongue base obstruction, as assessed by DISE prior to surgery, showed greater improvements in tongue base strength compared to those with severe obstruction. Further research is needed to confirm these findings

## Figures and Tables

**Table 1:** Baseline demographics and clinical characteristics of the enrolled participants.

Variables	Total	M Group	S Group	*p*
N	49	39	10	
Gender				1.000
Female	10 (20.4%)	8 (20.5%)	2 (20%)	
Male	39 (79.6%)	31 (79.5%)	8 (80%)	
Age, years old	38.3 ± 7.4	37.2 ± 7.2	42.6 ± 7.5	0.066
BMI, kg/m^2^	27.8 ± 3.9	28.4 ± 3.7	25.6 ± 4.3	0.050
Smoking history	12 (24.5%)	10 (25.6%)	2 (20%)	1

Abbreviation: BMI, body mass index; M group, patients with no or partial tongue base obstruction; S group, patients with severe tongue base obstruction.

**Table 2:** Comparison between the M group (patients with no or partial tongue base obstruction) and the S group (patients with severe tongue base obstruction).

Variables	Total	M Group	S Group	*p* Value
N	49	39	10	
AHI, events/h	36.0 ± 29.4	35.7 ± 28.1	37.0 ± 35.4	0.825
IOPI 1 month after surgery before training, kPa	27.6 ± 13.5	28.1 ± 13.7	25.6 ± 13.1	0.607
Training days	41.4 ± 14.3	41.3 ± 14.1	41.9 ± 16.0	0.901
IOPI after training, kPa	48.1 ± 13.0	50.5 ± 11.9	38.9 ± 13.7	0.010
Difference, kPa	20.6 ± 11.5	22.5 ± 11.8	13.3 ± 6.9	0.024

Abbreviation: AHI, apnea–hypopnea index; IOPI, Iowa Oral Performance Instrument; M group, patients with no or partial tongue base obstruction; S group, patients with severe tongue base obstruction.

**Table 3:** The univariate linear regression for the difference in tongue muscle power between pre-operation and post-training of the HEAL usage.

Variables	Estimate	S.E.	t Value	*p*
Male vs. female	0.49	4.12	0.12	0.905
Age, years	−0.06	0.22	−0.27	0.786
BMI, kg/m^2^	0.36	0.42	0.84	0.403
Smoking history	5.29	3.79	1.40	0.169
Alcohol history	1.12	5.49	0.21	0.839
Hypertension	−2.77	4.27	−0.65	0.52
Diabetes	−4.01	11.74	−0.34	0.734
AHI, events/h	0.04	0.06	0.67	0.507
M group vs. S group	9.12	3.90	2.34	0.024
Training days	−0.02	0.12	−0.21	0.833

Abbreviation: AHI, apnea–hypopnea index; BMI, body mass index; M group, patients with no or partial tongue base obstruction; S group, patients with severe tongue base obstruction.

**Table 4:** The different linear regression models for the difference in tongue muscle power between pre-operation and post-training of Healer usage.

Model	Covariate	Estimate (95%C.I.)	S.E.	t Value	*p* Value
Model 1	M group vs. S group	9.10 (−0.17, 18.37)	4.57	1.99	0.054
	Male vs. female	0.25 (−7.99, 8.49)	4.06	0.06	0.952
	Age, years	0.07 (−0.42, 0.57)	0.24	0.30	0.764
	BMI, kg/m^2^	−0.17 (−1.01, 0.67)	0.42	−0.41	0.682
	Smoking	0.13 (−8.27, 8.52)	4.13	0.03	0.976
	Training day	−0.003 (−0.25, 0.24)	0.12	−0.02	0.983
	intercept	13.76 (−19.65, 47.16)	16.45	0.84	0.409
Model 2	M group vs. S group	9.12 (0.14, 18.09)	4.43	2.06	0.047
	Male vs. female	0.24 (−7.83, 8.31)	3.98	0.06	0.953
	Age, years	0.08 (−0.39, 0.54)	0.23	0.33	0.746
	BMI, kg/m^2^	−0.17 (−1.00, 0.65)	0.41	−0.43	0.673
	Smoking	0.10 (−7.90, 8.10)	3.94	0.03	0.979
	intercept	13.62 (−16.91, 44.16)	15.05	0.91	0.372
Model 3	M group vs. S group	9.11 (0.27, 17.95)	4.36	2.01	0.044
	Male vs. female	0.24 (−7.72, 8.19)	3.92	0.06	0.952
	Age, years	0.08 (−0.38, 0.53)	0.23	0.34	0.738
	BMI, kg/m^2^	−0.17 (−0.98, 0.63)	0.40	−0.43	0.668
	intercept	13.58 (−16.32, 43.48)	14.76	0.92	0.363
Model 4	M group vs. S group	9.07 (0.27, 17.95)	4.24	2.14	0.039
	Age	0.07 (−0.37, 0.52)	0.22	0.34	0.738
	BMI	−0.17 (−0.96, 0.62)	0.39	−0.43	0.667
	intercept	13.83 (−16.32, 43.48)	13.99	0.99	0.329
Model 5	M group vs. S group	8.85 (0.58, 17.12)	4.11	2.15	0.037
	BMI, kg/m^2^	0.10 (−0.76, 0.95)	0.43	0.23	0.817
	intercept	10.80 (−12.25, 33.85)	11.45	0.94	0.351
Model 6	M group vs. S group	9.12 (1.27, 16.97)	3.90	2.34	0.024
	intercept	13.33 (6.33, 20.34)	3.48	3.83	<0.001

Abbreviation: BMI, body mass index; M group, patients with no or partial tongue base obstruction; S group, patients with severe tongue base obstruction.

## Data Availability

The data presented in this study are available on request from the corresponding author. The data are not publicly available due to privacy restrictions.

## Abbreviations

The following abbreviations are used in this manuscript:

AHI	Apnea–hypopnea index
BMI	Body Mass Index
CPAP	Continuous Positive Airway Pressure
DISE	Drug-induced sleep endoscopy
ENT	Ear, Nose, and Throat
IOPI	Iowa Oral Performance Instrument
ODI	Oxygen Desaturation Index
OSA	Obstructive sleep apnea
UPPP	Uvulopalatopharyngoplasty
VOTE	Velum (V), Oropharyngeal lateral walls (O), Tongue base (T), and Epiglottis (E)
